# Surge in antidepressant usage among adolescents and young adults during the COVID-19 pandemic: insights from an interrupted time series analysis

**DOI:** 10.1017/S2045796024000647

**Published:** 2024-11-07

**Authors:** Zeno Di Valerio, Daniela Fortuna, Marco Montalti, Lucia Alberghini, Anna Caterina Leucci, Alessio Saponaro, Elisa Sangiorgi, Elena Berti, Maurizia Rolli, Dario Tedesco

**Affiliations:** 1 Department of Innovation in Health and Social Services, Directorate-General for Health and Welfare, Emilia-Romagna Region, Bologna, Italy; 2 Department of Epidemiology, Mailman School of Public Health, Columbia University, New York, NY, USA; 3 Department of Biomedical and Neuromotor Sciences, University of Bologna, Bologna, Italy; 4 Azienda USL di Reggio Emilia, Reggio Emilia, Italy; 5 Department of Medicine and Surgery, University of Parma, Parma, Italy; 6 Department of Medical Affairs, Azienda USL di Piacenza, Piacenza, Italy

**Keywords:** antidepressant, COVID-19, depression, depressive disorder, pharmacoepidemiology

## Abstract

**Background:**

Depressive disorders are a major public health issue in Western societies, particularly among adolescents, young adults and women. The COVID-19 pandemic has exacerbated mental health challenges, increasing depression and anxiety symptoms, especially in younger people. This study focuses on the hard-hit Emilia-Romagna Region (ERR) in Italy, examining changes in antidepressant (AD) drug use post-COVID-19 to understand the pandemic’s effect on mental health.

**Methods:**

A population-based interrupted time series design and a segmented regression analysis was carried out on ERR pharmaceutical data (FED, direct dispensation pharmaceuticals, AFT, territorial pharmaceutical assistance) out to estimate changes in AD use during the three pandemic years (2020, 2021 and 2022) compared to 2017–2019.Analyses were stratified by age, gender, citizenship, population density of the area of residence.

**Results:**

A notable increase in AD consumption compared to what was expected was observed among younger age groups, and especially in females. In the 12–19 age group, a gradual increase was recorded from January 2021 until it reached +48% in 2022 (+58% among women, +30% among men). An even more remarkable growth in AD usage among non-Italian residents in the same age group was recorded compared to expected. A relevant increase, although smaller, was detected among individuals in the 20–34 age group, with a peak of +9% in 2022. These differences persisted up until the end of the observation period.

**Conclusions:**

The study suggests that the COVID-19 pandemic may have had a lasting negative impact on the mental health of younger individuals. The observed increase in AD use may foreshadow a potential long-term need for enhanced mental healthcare and services directed at this subpopulation.

## Background

In Western societies, depressive disorders represent a growing public health challenge, accounting for over one-third of disability-adjusted life years attributed to mental disorders and ranking as the second-highest contributor to years lived with disability worldwide (Institute for Health Metrics and Evaluation [IHME], 2020). This prevalence is even more pronounced among adolescents and young adults. In the European Union, the estimated affected population exceeded 22 million individuals, constituting 4.6% of the population in 2019. Notably, depressive disorders exhibit a higher prevalence among females compared to males (GBD 2019 Mental Disorders Collaborators, 2022).

The COVID-19 pandemic has introduced significant pressures on mental health by impacting various well-established determinants. Measures such as lockdowns, restrictions on social interactions, school closures and income instability have the potential to adversely affect mental well-being. The redirection of healthcare resources to combat the pandemic has further exacerbated these effects. A widespread increase in of depressive disorders during and after the pandemic has been well documented (COVID-19 Mental Disorders Collaborators, 2021). Symptoms of anxiety and depression experienced by young people have more than doubled in several European countries during this period (OECD/European Union, 2022). Italy has witnessed a noticeable deterioration in mental health indicators as well, especially among younger individuals and women, between 2019 and 2021 (ISTAT, 2022). Significant economic consequences must also be considered, as it is estimated that in Italy people with depression require three times the amount of days off work, and an estimated yearly cost of depressive disorders is 4–5 billion euros, of which 0.4 for pharmaceutical expenses in 2021 (Gruppo di lavoro, 2022; Ministero della Salute, 2022).

Monitoring indicators related to depressive disorders is crucial to understand the pandemic’s lasting societal and public health effects and consequently its implications for public welfare, mental health services and policy. Our study seeks to quantify the impact of the COVID-19 pandemic, along with associated restrictions and preventive measures, on depressive disorders in the Emilia-Romagna region (ERR), an Italian region with about 4.6 million inhabitants, hard hit by pandemic waves and among the largest consumers od antidepressant (AD) drugs in Italy (55.1 DDD/1000 inhabitants in 2021, compared to a national average of 44.6) (Osservatorio Nazionale sull’impiego dei Medicinali, 2023). Specifically, we aimed to do this by analyzing changes in AD dispensation by the National Health Service through an exploratory interrupted time series analysis.

## Methods

### Study design

We conducted a retrospective observational study to assess changes in AD dispensation trends after the COVID-19 pandemic onset. Segmented regression analysis was used to estimate trend shifts over time. The primary outcome was the daily prevalence of AD consumers, with daily incidence and DDDs per 1000 inhabitants as secondary outcomes.

### Data sources and population selection criteria

In Italy, all citizens are registered with the National Health Service and the Italian regions are responsible for the organization and management of publicly funded healthcare services, including the provision of drugs, for their residents. Using an anonymized identifier attributed to each assisted resident, the individual consumption of AD drugs was identified through record-linkage between the registry of individuals assisted by the Regional Health Service and the regional databases of dispensed drugs (AFT, territorial pharmaceutical assistance, and FED, direct dispensation pharmaceuticals, with data on drugs dispensed by pharmacies and directly dispensed by community health authorities, respectively). In these, all drugs dispensed to ERR residents are reported, with Anatomical Therapeutic Chemical (ATC) codes, DDD number and date of dispensation. ADs were defined as belonging to the N06A subgroup of the ATC classification system.

To assess changes in the use of AD drugs after the COVID-19 pandemic, compared to the previous 3 years, we selected residents in the ERR who were dispensed AD drugs between 1 January, 2017, and 31 December, 2022. While we acknowledge medicine dispensation does not correspond to actual intake, we will refer to individuals being dispensed ADs as ‘AD users’ as a simplification.

### Endpoints and statistical analysis

The effects on AD use of the COVID-19 pandemic and the restriction associated with it were measured considering three different endpoints, assessed daily and monthly: prevalence and incidence of AD consumers, and DDDs per 1000 inhabitants.

**Figure 1.**
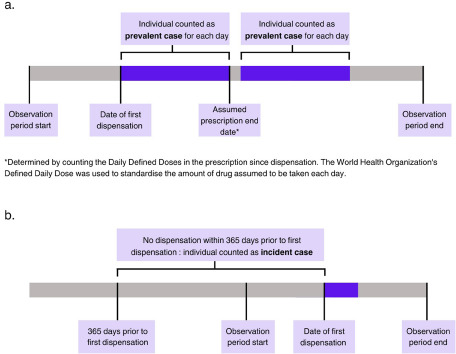
Diagram outlining inclusion criteria as prevalent (a) and incident (b) cases.

The daily prevalence of AD consumers was estimated by counting everyone who was considered as being under treatment for that day. Individuals were considered as under treatment from the date of first dispensation for a number of days equal to the DDDs prescribed. For each day of treatment, the subject was counted as a prevalent case. The daily frequency of new AD consumers was estimated counting the individuals with a dispensation of AD drugs without any registered dispensation in the 365 days prior (Fig. 1).

The above endpoints, stratified by age, gender, citizenship and population density of residence area, were explored during the three pandemic years considered (2020, 2021 and 2022) and compared with the expected numbers estimated from the pre-pandemic period 2017–2019. Age strata were defined combining common definitions of human psychological development stages (e.g. childhood, young adulthood, adulthood, old age) and social setting changes within the Italian system (e.g. age at high school graduation).

Population density of the area of residence (city, small town, rural) was defined according to Eurostat’s Methodological manual on territorial typologies (Eurostat, 2019). For each endpoint, a descriptive analysis of the trend was first performed from 1 January 2017 to 31 December 2022 to assess the seasonal and cyclical components. A population-based interrupted time series design and a segmented regression analysis was carried out using Poisson or negative binomial models, with the logarithm of the daily living population, weighted by age and gender as offset, and the time trend, the seasonal component expressed by sine and cosine functions of the trend, the days of the week and the months as covariates (Bernal *et al.*, 2020; Bhaskaran et al., 2013; Wagner *et al.*, 2002). The models were performed on 2017–2019 data related to each type of endpoint and the resulting parameters were applied to the 2020, 2021 and 2022 data, to estimate the expected frequency based on the trend of the 3 years pre-pandemic period.

The ability of the models to reduce autocorrelation was evaluated using the Ljung-Box test (Ljung and Box, 1978). For each endpoint, the observed and expected trends were graphically represented over the 3 years of the COVID-19 pandemic, highlighting three pandemic waves: from March to April 2020, corresponding to the nationwide lockdown; from October 2020 to May 2021; and from October to December 2021.

The monthly incident rate ratio (IRR) between the number of observed and expected AD consumers for each endpoint was evaluated through a further Poisson or negative binomial model, similar to the one described above but with the addition of the interaction between month and year as a dichotomous variable, among the covariates. The monthly IRR was obtained from the exponential of the coefficient estimated for the dichotomous variable indicating the interaction between month and year (Wagner *et al.*, 2002). Positive or negative IRR values respectively indicate an excess, or a reduction compared to the same months of the previous 3-year period. To maintain the 2017–2019 3-year period as the reference time for all estimates, the measures observed in 2021 and 2022 were excluded from the model for the 2020 IRR estimates, as was done for the models applied to estimate the IRRs for 2021 and for 2022.

The monthly IRRs for each endpoint are presented graphically using forest plots, alongside the time trend graphs, and the values are reported in the text as percentages obtained by multiplying the IRR minus 1 by 100: (IRR − 1) × 100.

All analyses were performed using R version 3.6.3 (The R Foundation for Statistical Computing, Wien) and SAS version 9.3 (SAS Institute, Inc. Cary, NC, USA).

## Results

Between 2017 and 2022, the yearly consumers of AD in ERR varied from a minimum of 336,592 in 2017 to a maximum of 345,587 in 2022. Their demographic characteristics remained largely constant over time during the study period (Table 1).

**Table 1.** Demographic characteristics and baseline data of study participants, yearly prevalence and incidence of AD use (2017–2022)

	Year											
	2017				2018				2019			
	*n*	%	Prevalence %	Incidence %	*n*	%	Prevalence %	Incidence %	*n*	%	Prevalence %	Incidence %
Age group												
0–11	50	<0.1	<0.1	<0.1	39	<0.1	<0.1	<0.1	38	<0.1	<0.1	<0.1
12–19	1,703	0.5	0.5	0.3	1,901	0.6	0.6	0.4	1,997	0.6	0.6	0.4
20–34	15,237	4.5	2.4	1.1	15,542	4.5	2.5	1.2	15,959	4.6	2.5	1.3
35–44	27,684	8.2	4.9	1.7	26,975	7.9	4.9	2.1	25,741	7.5	4.9	2.2
45–64	105,068	31.2	8.0	2.0	107,621	31.4	8.1	3.0	108,562	31.6	8.0	3.2
65–74	60,413	17.9	12.2	2.6	61,353	17.9	12.3	4.3	61,725	17.9	12.2	4.5
75+	126,437	37.6	20.9	5.0	129,053	37.7	21.3	7.9	129,904	37.8	21.3	8.2
Sex												
Females	231,727	68.8	10.1	2.5	235,895	68.9	10.3	3.8	236,594	68.8	10.3	4.0
Males	104,865	31.2	4.9	1.4	106,589	31.1	4.9	2.0	107,332	31.2	5.0	2.1
Citizenship												
Italian	325,907	96.8	8.1	2.1	330,324	96.4	8.3	3.1	330,968	96.2	8.3	3.3
Other	10,685	3.2	2.5	0.2	12,160	3.6	2.7	0.3	12,958	3.8	2.7	0.3
Area of residence												
City	121,539	36.1	7.7	2.0	123,783	36.1	7.8	3.0	124,653	36.2	7.8	3.1
Small town	110,692	32.9	7.4	1.9	112,332	32.8	7.5	2.8	113,124	32.9	7.6	3.0
Rural area	104,361	31	7.7	2.1	106,369	31.1	7.9	3.0	106,149	30.9	7.9	3.1
Total AD consumers	336,592	100.0	7.6	2.0	342,484	100.0	7.7	2.9	343,926	100.0	7.7	3.1

	Year											
	2020				2021				2022			
	*n*	%	Prevalence %	Incidence %	*n*	%	Prevalence %	Incidence %	*n*	%	Prevalence %	Incidence %
Age group												
0–11	38	<0.1	<0.1	<0.1	43	<0.1	<0.1	<0.1	42	<0.1	<0.1	<0.1
12–19	1,965	0.6	0.6	0.4	2,705	0.8	0.8	0.6	3,312	1	1.0	0.7
20–34	15,470	4.6	2.4	1.2	16,911	4.9	2.6	1.4	18,066	5.2	2.7	1.5
35–44	23,670	7.0	4.7	2.1	23,304	6.8	4.7	2.3	22,236	6.4	4.6	2.3
45–64	107,179	31.7	7.9	3.1	109,124	31.6	7.9	3.3	108,273	31.3	7.8	3.3
65–74	62,179	18.4	12.1	4.6	62,542	18.1	12.2	4.9	61,875	17.9	12.1	4.8
75+	128,098	37.8	21.1	8.4	130,527	37.8	21.3	8.9	131,783	38.1	21.3	8.9
Sex												
Females	232,547	68.7	10.2	4.0	237,706	68.9	10.4	4.3	238,067	68.9	10.4	4.3
Males	106,052	31.3	4.9	2.1	107,450	31.1	5.0	2.2	107,520	31.1	5.0	2.3
Citizenship												
Italian	325,833	96.2	8.2	3.3	331,492	96	8.4	3.6	331,237	95.8	8.5	3.6
Other	12,766	3.8	2.7	0.3	13,664	4	2.7	0.3	14,350	4.2	2.7	0.4
Area of residence												
City	122,854	36.3	7.7	3.2	125,373	36.3	7.8	3.4	126,140	36.5	7.9	3.4
Small town	111,295	32.9	7.5	3.0	114,437	33.2	7.7	3.3	114,627	33.2	7.7	3.3
Rural area	104,450	30.8	7.8	3.2	105,346	30.5	7.9	3.3	104,820	30.3	7.8	3.3
Total AD consumers	338,599	100.0	7.6	3.1	345,156	100.0	7.8	3.3	345,587	100.0	7.8	3.3

Approximately 84% of AD consumers were over 45 years old, with 31% between 45 and 64, 18% between 65 and 74 and 38% over 75. Despite being a minority of the total AD consumer population, younger age brackets showed notable increases as a proportion of total consumers, with a rise from 0.5% in 2017 to 1% in 2022 in the 12–19, and from 4.5% to 5.2% in the 20–34 age strata. Gender differences persisted over time, with around 69% of AD consumers being female. A large majority was of Italian nationality, at about 96%. When looking at population density, 36% lived in urban areas, 33% in small towns and about 30% in rural areas.

Prevalence of AD use increased from 7.6% in 2017 to 7.8% in 2022. New AD users slightly increased especially in the last 2 years from 2% to 3.3%. The greatest change was recorded in the 12–19 and 20–34 age groups, in both prevalence and incidence rate.

**Figure 2.**
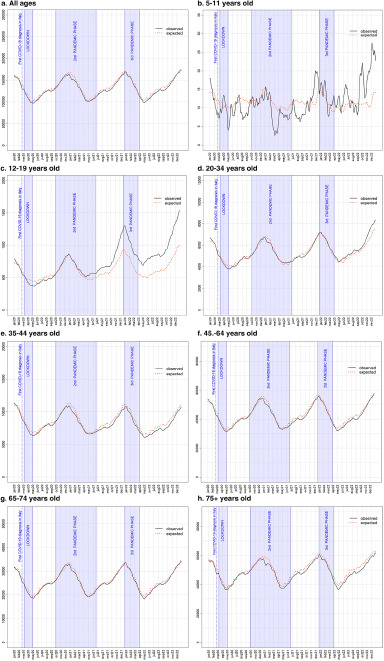
Comparison between expected daily prevalence of AD consumers and the 2017–2019 trend in the observation period (January 2020–December 2022).

The interrupted time series analysis allowed a more accurate evaluation of these excesses throughout the three main pandemic phases (Fig. 2). The comparison between the observed number of daily AD users from 2020 to 2022 to the trend in previous years did not show remarkable differences (Fig. 2a). However, among children aged 0–11 years (Fig. 2b) there was a surge in the trend in the last 4 months of 2022, although it should be emphasized that these were less than 25 cases. A more reliable and notable deviation from the expected trend was found among adolescents (12–19), with onset from March 2021 and progressive widening over time until December 2022 (Fig. 2c). A smaller although significant increase in AD consumption occurred among young adults (20–39), starting from the last half of 2022 (Fig. 2d).

For other age groups, no significant differences were found compared to the expected values, not even when stratifying by sex, and population density of the place of residence (Figure 2e–g, Supplementary Table 2). A largely lower than expected number of AD consumers can be observed among residents with foreign nationality starting from March 2020, with differences stably exceeding −20% from April 2022 onwards, and an all-time low of −23.7% in May 2022 (Supplementary Table 2).

### Adolescents (12–19)

**Figure 3.**
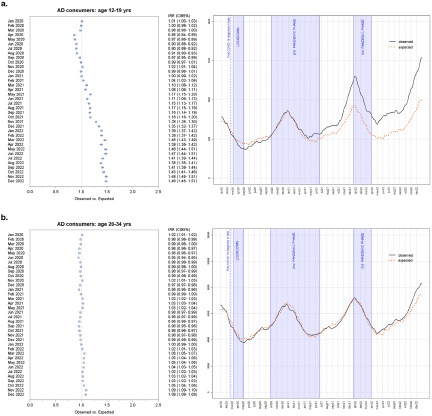
Forest plot of monthly IRRs of observed versus expected AD consumers, and daily differences among all AD consumers aged 12–19 (a) and 20–34 (b).

The largest increase in AD consumption was registered among the 12–19 age group (Fig. 3a). After a temporary reduction in AD usage in the first months of the pandemic, during the lockdown and the summer months that followed, with the advent of the second pandemic wave consumption began to grow, exceeding the expected value from February 2021 onwards. In the last 2 months of 2021, there was a surge in AD consumption which reached its highest value in January 2022, with a +39.3% excess. The excess number of AD consumers did not decrease in the following months and worsened after the third pandemic phase, reaching +48% in the last 2 months of 2022 (Fig. 3a). Incidence substantially followed the same pattern of prevalence. During the lockdown phase, a reduction of around 50% of new AD consumers took place. Incidence was higher than expected in May 2021 (+31.9%), during the second pandemic wave and then in August (+46.8%). The largest number of new AD consumers was recorded close to the start of third pandemic phase: in November 2021 (+29.8%) and in December 2021 when 315 new cases were recorded, +55.4% compared to expected. The excess of new AD consumers continued in 2022, reaching significant high values in February (+48.9%), April (+36.8%) and June (+36.3%) (Supplementary Table 3).

Similarly to what was observed among the general population, more than 60% of AD consumers among adolescents were females. The gap between the two sexes widened during the pandemic. In fact, among adolescent females an increase of 10% compared to the expected value began to be recorded from March 2021 onwards, during the second pandemic wave. The excess gradually grew over time until it reached +60% in June 2022 and persisted up to the last months of the study period: +57.5% and +54.2% in November and December 2022, respectively. On the other hand, among adolescent males, the first sign of greater AD consumption was recorded as early as January 2021 (+4%), until April 2021 (+6%). The excess of AD use ensued again starting from November 2021 and continued up until the end of 2022, reaching a maximum of 30% in February 2022, during the third pandemic wave.

The growth of AD usage among adolescents of non-Italian citizenship deserves mentioning, as an excess between 20% and 30% was recorded in the first 3 months of 2020. From October 2020 onwards the increase in AD consumption resumed, continuing until the end of the study period and more than doubling in March 2022 (+109%).

**Figure 4.**
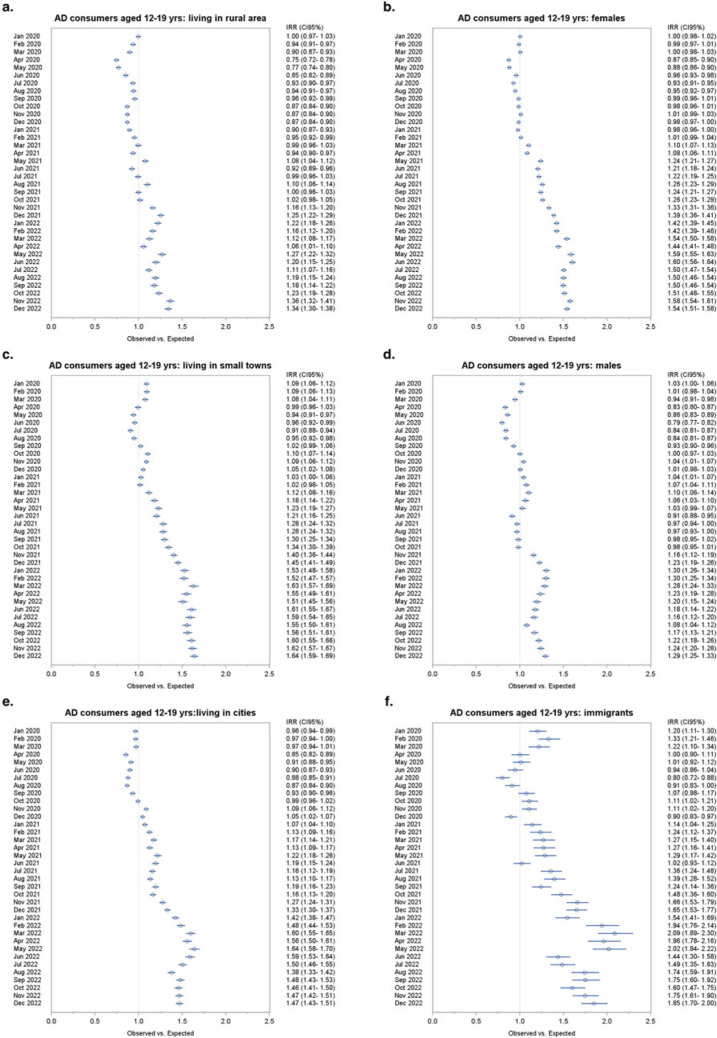
Forest plots of monthly IRR of observed AD consumers ages 12–19 versus expected based on the 2017–2019 trend, by subpopulation.

Differences in trends were observed in relation to the population density of the area of residence: the increase in AD consumption was less prominent among adolescents living in rural areas than those living in cities and small towns (Fig. 4).

### Young adults (20–34)

Albeit to a lesser extent, a greater AD use was also recorded among young adults in the 20–34 age group during the 3 years of the pandemic, compared to 2017–2019 (Fig. 3b). The first excesses were recorded at the beginning and at the end of the second pandemic phase, with increases of around 2–3%. A growing trend began in February 2022 and lasted until the end of the study period. The increase peaked in the last 2 months of 2022 with a +9.4% in November and a +8.7% in December 2022 (Fig. 3b).

Distinguishing by gender, the pandemic seems to have had an opposite impact in males and females. Among female young adults, a 1–2% higher number of AD consumers was recorded from July 2020 onwards, with some fluctuations in the following months. From November 2021, the excess gradually worsened, reaching +14–15% during the third pandemic phase. The largest increases compared to expected were +18.8% and 18.5% in November and December 2022, respectively. In contrast, a less than predicted number of male AD consumers was observed. From a low point of −10.8% in January 2022, the gap slowly narrowed towards reaching the end of the study period, with −2.3% and −3.4% registered in November and December 2022, respectively.

The amount of AD consumers of foreign nationality in this age group was lower than predicted, with the first negative differences appearing in March 2020, and reaching a minimum of −27.5% in August 2021. The negative gap persisted up until the last months of the study period.

**Figure 5.**
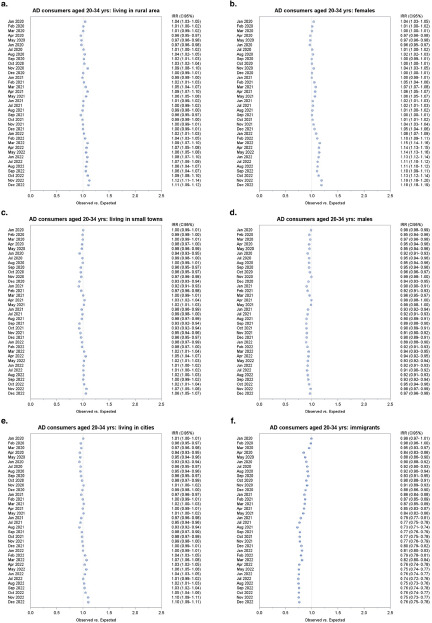
Forest plots of monthly IRR of observed AD consumers ages 20–34 versus expected based on the 2017–2019 trend, by subpopulation.

In cities and rural areas, the excess of AD consumers aged 20–34 mirrored what happened in the whole age group. On the other hand, during the three pandemic phases no significant differences compared to expected were recorded in small towns, except for excesses of +6.6% and +5.6% recorded in November and December 2022, respectively (Fig. 5).

### Other age groups

Within the 35–44 age group, differences from the pre-pandemic trend were smaller and stably negative throughout the study period, with a minimum −8.2% difference recorded in October 2022. Similarly, adults aged 55–64 only showed negative differences in comparison with the pre-pandemic trend, peaking at −5.2% in August 2022. Only during November 2020, March 2021 and February and March 2022 small positive differences were recorded, with a peak of +2.2% in March 2021. Young-older adults (65–74) showed the smallest differences, with a low point of −3.6% in June 2021. Positive differences, albeit small, were recorded during February, March and April 2020, January, February and March 2021 and November 2022, with a positive peak in March 2021 of +3.0%. Among old-older adults (75+) a minimum −6.9% difference was recorded in July 2022 (Supplementary Table 1). Only during the month of March 2021 a positive difference of +0.7% was recorded. Within these age groups, negative differences were recorded nearly throughout the whole study period, with <1% sex differences.

Data on the trends for incidence of new AD consumers and DDD/1000 inhabitants dispensed between 2017 and 2022 can be found in Supplementary Table 3.

## Discussion

Our findings indicate an impact of the COVID-19 pandemic and its socio-economic consequences on AD use among young individuals. This trend persisted throughout the pandemic, demonstrating a striking increase of up to +50% among the 12–19 age group and up to +10% among the 20–34 age group. Evidence on AD prescription and dispensation trends during the pandemic is limited, particularly concerning specific subpopulations. A Canadian study focusing on children and young subjects reported significant increases in prescriptions for antianxiety and antidepression medications among individuals aged 10–14, corroborating our findings (Daneshmand *et al.*, 2023). If AD dispensation may be interpreted as a proxy for depressive disorders, our results are consistent with other data sources indicating increased prevalence of depressive disorders and trends in AD dispensation in Italy (Ministero della Salute, 2022; Osservatorio Nazionale sull’impiego dei Medicinali, 2023). The connection between deteriorated mental health indicators, such as those observed in our study, and an actual increase in depressive disorders is subject to debate. While evidence since 2020 indicates a clinical impact of the pandemic on depressive disorders, particularly among younger demographics (Kauhanen *et al.*, 2023), a corresponding escalation in suicide rates is not definitely evident or consistent, as demonstrated by a recent systematic review (Pirkis *et al.*, 2021). This apparent inconsistency may stem from earlier diagnosis due to heightened awareness on mental health conditions and increased accessibility of related services. It also may reflect a delayed effect of the pandemic on suicide incidence, as it could require considerable time, perhaps years, for an effect on such an outcome to manifest itself.

One of the reasons behind the observed changes among the school-age group might be found in the large disruption to education the pandemic has caused. The United Nations Educational, Scientific and Cultural Organization (UNESCO) declared COVID-19 to be the most severe disruption to global education in history, estimating 1.6 billion learners in over 190 countries were fully or partially out of school in 2020 (UNESCO, 2024). With school closures and wider social restrictions in place, young people have been unable to come together in physical spaces, affecting their ability to learn and interact with peers.

When considering young adults, the repercussions of job loss, income instability and the financial strain among those who managed to retain their employment may have exerted a similar influence on mental health outcomes (De Miquel *et al.*, 2022). Substantial evidence shows the correlation between economic adversity and mental well-being, especially among young adults (Adams *et al.*, 2016; Ganson *et al.*, 2021), who are also disproportionately affected by unemployment during and in the aftermath of economic downturns compared to older demographics (Frasquilho *et al.*, 2016). In addition to financial distress, it is possible to suggest an overall traumatic influence of the pandemic measures on younger individuals, or an acceleration of other social processes associated with worsened mental health, such as the prolonged use of electronic devices and social networks (Santos *et al.*, 2023), the reduction in face-to-face social activities and impaired building of strong social structures in a crucial phase of development (Orben *et al.*, 2020).

Female gender showed a larger increase in AD dispensation across the study period among individuals aged 12–19. Within the 20–34 age group, women accounted for the whole observed increase, while men showed negative variations compared to the pre-pandemic period. Women have been consistently shown to be more exposed to depressive disorders (Kessler, 2003). However, this does not explain the observed acceleration from the pre-pandemic trend. Women have also been shown to be particularly exposed to the consequences of pandemic: lower salaries, less savings and less secure employment compared to their male counterparts made them more likely to be financially disadvantaged (Burki, 2020; Wenham *et al.*, 2020). Additionally, women are at higher risk of domestic violence, which has shown an increase during periods of lockdown and stay-at-home orders (Arenas-Arroyo *et al.*, 2021; Piquero *et al.*, 2021). Furthermore, in Italy they have been shown to bear a disproportionate amount of unpaid work, notably in terms of caring for family members, when compared to men. Suggestively, women still contribute to 65.7% of the time spent by Italian households on caring for children and/or other family members (Istituto Nazionale di Statistica, 2023). The added strain of caregiving duties, compounded by limited non-COVID healthcare services, likely had a greater effect on women’s mental well-being during the pandemic.

Non-Italian citizens exhibited distinct consumption patterns, with an overall lower-than-expected prevalence of AD use. However, within the younger cohorts, those of school age showed an elevated AD use relative to their Italian counterparts. In contrast, working-age individuals demonstrated a notably reduced AD consumption rate. These divergent findings may reflect variations in access to mental health services between different age groups. For instance, the school environment potentially facilitates the identification and management of mental health issues in younger individuals through educator oversight. Meanwhile, foreign-born young adults might encounter distinct barriers to access to mental health services that have to do with the difficulties in navigating the Italian healthcare system, especially when considering the additional challenges posed by the COVID-19 pandemic. This diminished engagement with the Italian healthcare system among foreign nationals could partly explain their lower AD use. Literature seems to confirm these observations: barriers to care for non-Italian adults influence their ability to seek medical help, thereby causing a lower access to community health services and mental health services (De Luca *et al.*, 2013; Spinogatti *et al.*, 2015). Differences in language proficiency, and consequently in the ability to express psychological distress, between adolescents and young adults of foreign nationality may also have contributed to the observed differences. These observations make it imperative for the healthcare system to proactively address the needs of this population.

Our findings show no relevant differences in AD consumers between the pre-pandemic period and the 2020–2022 period, in the general population and even negative differences in older age groups in comparison with the pre-pandemic trend. While possibly explained by the severe disruptions to primary care services caused by COVID-19, these findings are in contrast with other studies employing a similar design, which show an increase in AD usage in 2020 (Frangou *et al.*, 2023; Pazzagli *et al.*, 2022). On the other hand, a study conducted in Slovenia on AD drug concentration in wastewater did not find relevant changes in relation to the pandemic (Laimou-Geraniou *et al.*, 2023). To our knowledge however, no studies investigated differences later than 2020, a rather exceptional year during which changes in AD usage might have been substantially influenced by the efficiency of non-COVID-19 related health services rather than by actual changes in therapeutic needs. Our study has some notable limitations. As is bound to happen with database studies, we measured drug dispensation and not compliance with therapy. While dispensation data are arguably more adherent to actual consumption than prescription data, they still does not reflect medication uptake. Moreover, it must be pointed out that changes in AD use do not necessarily reflect underlying epidemiological shifts in clinical manifestations. Especially at the pandemic’s onset, they are likely to have been strongly influenced by availability of psychiatric services, and later they may reflect changes in awareness among individuals, parents, peers and institutions about psychiatric symptoms and help-seeking. Prescriptions dispensed to individuals residing in institutional and elderly care settings were not accessible and are therefore not included in our analysis. This omission is noteworthy, because a more severe impact on mental health may be expected in this subpopulation both because of the disease and its social consequences (Armitage and Nellums, 2020). Additionally, our study excluded individuals who were not enrolled in the regional healthcare system, such as Italians temporarily residing in the region for study or work or undocumented foreign nationals. Moreover, it should be noted that DDDs and derived measures are not reliable measures of drug consumption in children, due to their definition as average maintenance doses of a drug for its main indication in adults. Dosage in children may vary according to age and weight.

## Conclusions

Our study contributes to the growing body of knowledge demonstrating the detrimental impact of the COVID-19 pandemic on the mental health of younger individuals. It is particularly notable that women and people of foreign citizenship appear to be the most affected, and how this impact seems to have lingering consequences. Indeed, the observed changes in AD usage did not diminish even as the pandemic phase concluded and long after restrictions were lifted. This should prompt close monitoring of mental health indicators to confirm whether our observations and those of others will translate into an increased demand for mental health services in the future. As the hardships caused by the COVID-19 pandemic slowly fade from collective memory and public debate, healthcare workers and decision-makers should consider whether we can expect such an age-defining event to influence mental healthcare for the years to come, and how to make use of this experience in enhancing preparedness and informing future pandemic response plans.

## Supporting information

Di Valerio et al. supplementary material 1 Di Valerio et al. supplementary material

Di Valerio et al. supplementary material 2 Di Valerio et al. supplementary material

## Data Availability

Data can be made available at the authors’ discretion upon motivated request sent to the corresponding author.

## Supplementary material

The supplementary material for this article can be found at https://doi.org/10.1017/S2045796024000647.
