# Supplementary material for: Surge in antidepressant usage among adolescents and young adults during the COVID-19 pandemic: insights from an interrupted time series analysis
Source: Epidemiol Psychiatr Sci. 2024 Nov 7;33:e62. doi: 10.1017/S2045796024000647 (PMC11561682; doi:10.1017/S2045796024000647)
Supplement: Di Valerio et al. supplementary material 2 — Di Valerio et al. supplementary material [file S2045796024000647sup002.docx]

**SUPPPLEMENTARY MATERIAL**

**Supplementary Table 1:** Monthly number of AD consumers in the observation period (2020-2022) for each age group, and variations from the 2017-2019 trend expressed in percentage.

|  |  | **All** | | **0-11** | | **12-19** | | **20-34** | | **35-44** | | **45-64** | | **65-74** | | **75+** | |
| --- | --- | --- | --- | --- | --- | --- | --- | --- | --- | --- | --- | --- | --- | --- | --- | --- | --- |
| **Year** | **Month** | **N** | **(IRR-1)% (CI 95%)** | **N** | **(IRR-1)% (CI 95%)** | **N** | **(IRR-1)% (CI 95%)** | **N** | **(IRR-1)% (CI 95%)** | **N** | **(IRR-1)% (CI 95%)** | **N** | **(IRR-1)% (CI 95%)** | **N** | **(IRR-1)% (CI 95%)** | **N** | **(IRR-1)% (CI 95%)** |
| **2020** | **January** | 215,677 | -0.8 (-0.9- -0.7) | 24 | 2.7 (-8.3- 15.1) | 1,003 | 1.2 (-0.5- 2.9) | 8,697 | 1.6 (1.0- 2.1) | 14,760 | -1.1 (-1.6- -0.7) | 69,815 | -0.3 (-0.5- -0.1) | 41,447 | -2.0 (-2.3- -1.8) | 79,931 | -1.1 (-1.3- -0.9) |
|  | **February** | 196,525 | -2.0 (-2.1- -1.8) | 16 | -23.9 (-34.0- -12.4) | 876 | -0.2 (-2.1- 1.7) | 7,847 | -1.6 (-2.2- -1.0) | 13,194 | -1.3 (-1.8- -0.9) | 63,605 | -1.1 (-1.4- -0.9) | 37,533 | -2.7 (-3.0- -2.4) | 73,454 | -2.8 (-3.0- -2.6) |
|  | **March** | 180,112 | -1.0 (-1.1- -0.9) | 20 | 1.8 (-11.0- 16.4) | 712 | -1.8 (-3.7- 0.2) | 6,789 | -1.1 (-1.7- -0.5) | 11,679 | -2.1 (-2.5- -1.6) | 57,258 | -0.8 (-1.1- -0.6) | 34,046 | -1.9 (-2.2- -1.6) | 69,608 | -0.7 (-0.9- -0.5) |
|  | **April** | 156,835 | -2.1 (-2.3- -2.0) | 16 | -11.7 (-23.1- 1.4) | 611 | -13.9 (-15.7- -12.0) | 5,941 | -3.9 (-4.5- -3.2) | 10,008 | -5.0 (-5.4- -4.5) | 49,956 | -2.2 (-2.4- -2.0) | 29,748 | -1.5 (-1.9- -1.2) | 60,555 | -1.5 (-1.7- -1.3) |
|  | **May** | 152,617 | -3.0 (-3.1- -2.9) | 15 | -19.5 (-30.5- -6.9) | 589 | -12.6 (-14.5- -10.7) | 5,742 | -3.6 (-4.2- -2.9) | 9,703 | -5.1 (-5.6- -4.6) | 48,154 | -3.3 (-3.5- -3.1) | 28,960 | -2.8 (-3.1- -2.5) | 59,454 | -2.2 (-2.4- -2.0) |
|  | **June** | 161,468 | -3.2 (-3.3- -3.1) | 19 | -12.8 (-23.8- -0.2) | 624 | -9.8 (-11.7- -7.9) | 5,924 | -5.5 (-6.2- -4.9) | 10,171 | -4.4 (-4.9- -3.9) | 50,666 | -2.9 (-3.2- -2.7) | 31,115 | -3.3 (-3.5- -3.0) | 62,949 | -2.8 (-3.0- -2.6) |
|  | **July** | 173,656 | -2.3 (-2.4- -2.2) | 20 | -12.4 (-22.8- -0.6) | 661 | -9.9 (-11.7- -8.0) | 6,548 | -1.7 (-2.3- -1.1) | 10,941 | -2.2 (-2.7- -1.7) | 54,438 | -2.5 (-2.7- -2.2) | 33,446 | -2.5 (-2.8- -2.2) | 67,602 | -1.9 (-2.1- -1.7) |
|  | **August** | 172,292 | -2.1 (-2.2- -2.0) | 17 | -35.2 (-43.9- -25.1) | 659 | -8.7 (-10.5- -6.9) | 6,601 | -0.9 (-1.6- -0.3) | 10,845 | -3.1 (-3.5- -2.6) | 54,191 | -2.5 (-2.7- -2.3) | 33,158 | -1.7 (-2.0- -1.5) | 66,821 | -1.8 (-2.0- -1.6) |
|  | **September** | 179,729 | -2.2 (-2.3- -2.1) | 18 | -12.0 (-23.0- 0.5) | 734 | -3.1 (-5.0- -1.2) | 6,894 | -2.2 (-2.8- -1.5) | 11,494 | -2.3 (-2.8- -1.8) | 56,961 | -1.5 (-1.7- -1.3) | 34,905 | -1.6 (-1.9- -1.4) | 68,723 | -3.0 (-3.1- -2.8) |
|  | **October** | 195,802 | -1.2 (-1.4- -1.1) | 22 | -4.1 (-15.3- 8.7) | 881 | -0.9 (-2.6- 0.9) | 7,597 | -1.3 (-1.8- -0.7) | 12,728 | -1.0 (-1.4- -0.6) | 62,600 | -0.8 (-1.0- -0.6) | 38,288 | -0.5 (-0.8- -0.2) | 73,686 | -2.0 (-2.2- -1.8) |
|  | **November** | 209,462 | 0.8 (0.7- 0.9) | 18 | 4.3 (-8.2- 18.5) | 1,038 | 2.2 (0.5- 4.0) | 8,309 | 1.9 (1.3- 2.5) | 13,880 | -0.3 (-0.7- 0.2) | 68,038 | 1.5 (1.3- 1.7) | 41,176 | 1.9 (1.6- 2.1) | 77,003 | -0.0 (-0.2- 0.2) |
|  | **December** | 212,495 | -3.2 (-3.3- -3.1) | 23 | -12.8 (-22.4- -1.9) | 1,114 | -0.6 (-2.1- 1.0) | 8,657 | -2.6 (-3.1- -2.0) | 14,050 | -4.6 (-5.0- -4.2) | 69,646 | -2.7 (-2.9- -2.5) | 41,935 | -2.5 (-2.7- -2.2) | 77,070 | -3.6 (-3.7- -3.4) |
| **2021** | **January** | 207,208 | -5.2 (-5.3- -5.1) | 23 | -1.3 (-12.9- 11.8) | 1,055 | 0.2 (-1.5- 2.0) | 8,453 | -4.3 (-4.9- -3.8) | 13,513 | -6.7 (-7.1- -6.3) | 67,496 | -4.2 (-4.4- -4.0) | 40,441 | -3.6 (-3.9- -3.4) | 76,227 | -6.5 (-6.7- -6.4) |
|  | **February** | 194,969 | -1.0 (-1.2- -0.9) | 15 | -40.1 (-49.2- -29.3) | 970 | 3.6 (1.6- 5.7) | 7,906 | -0.7 (-1.4- -0.1) | 12,305 | -3.1 (-3.6- -2.6) | 63,054 | -0.4 (-0.7- -0.2) | 37,619 | 0.1 (-0.2- 0.4) | 73,100 | -1.7 (-2.0- -1.5) |
|  | **March** | 185,678 | 1.5 (1.4- 1.7) | 12 | -54.4 (-62.4- -44.9) | 877 | 10.0 (7.8- 12.2) | 7,410 | 2.2 (1.5- 2.9) | 11,452 | -1.2 (-1.7- -0.7) | 59,507 | 2.2 (2.0- 2.4) | 35,420 | 3.0 (2.7- 3.3) | 71,000 | 0.7 (0.5- 0.9) |
|  | **April** | 163,657 | -0.7 (-0.8- -0.5) | 16 | -15.0 (-26.8- -1.4) | 843 | 8.2 (5.9- 10.5) | 6,801 | 3.3 (2.6- 4.0) | 10,346 | -0.7 (-1.2- -0.1) | 52,474 | -0.1 (-0.3- 0.2) | 30,912 | 0.8 (0.4- 1.1) | 62,265 | -2.2 (-2.5- -2.0) |
|  | **May** | 157,583 | -2.2 (-2.3- -2.0) | 14 | -19.9 (-31.4- -6.5) | 852 | 17.4 (15.0- 19.8) | 6,487 | 2.8 (2.0- 3.5) | 9,859 | -0.9 (-1.4- -0.3) | 49,842 | -1.5 (-1.7- -1.2) | 29,583 | -2.0 (-2.3- -1.7) | 60,946 | -3.6 (-3.9- -3.4) |
|  | **June** | 163,420 | -4.5 (-4.7- -4.4) | 14 | -21.2 (-32.0- -8.5) | 839 | 11.1 (8.8- 13.4) | 6,563 | -2.0 (-2.7- -1.3) | 9,979 | -4.9 (-5.4- -4.3) | 50,992 | -4.8 (-5.0- -4.6) | 31,288 | -3.6 (-3.9- -3.3) | 63,745 | -5.0 (-5.2- -4.8) |
|  | **July** | 171,749 | -4.5 (-4.6- -4.3) | 15 | -25.7 (-35.6- -14.3) | 907 | 14.7 (12.5- 17.0) | 6,802 | -2.2 (-2.8- -1.5) | 10,312 | -6.5 (-7.0- -6.1) | 53,411 | -5.2 (-5.5- -5.0) | 33,010 | -2.6 (-2.9- -2.3) | 67,292 | -4.7 (-4.9- -4.5) |
|  | **August** | 171,687 | -4.0 (-4.1- -3.8) | 13 | -37.5 (-46.5- -27.0) | 915 | 16.9 (14.7- 19.2) | 6,794 | -3.8 (-4.4- -3.1) | 10,294 | -6.5 (-7.0- -6.0) | 53,659 | -4.6 (-4.8- -4.4) | 33,105 | -1.8 (-2.1- -1.6) | 66,907 | -4.2 (-4.4- -4.0) |
|  | **September** | 177,639 | -4.5 (-4.6- -4.4) | 17 | -20.5 (-31.3- -7.9) | 986 | 16.4 (14.2- 18.7) | 7,054 | -4.4 (-5.1- -3.8) | 10,791 | -6.2 (-6.7- -5.8) | 55,645 | -4.8 (-5.0- -4.6) | 34,231 | -2.7 (-3.0- -2.4) | 68,915 | -5.0 (-5.2- -4.8) |
|  | **October** | 191,348 | -4.1 (-4.2- -4.0) | 23 | -5.0 (-17.0- 8.7) | 1,140 | 17.7 (15.6- 19.9) | 7,717 | -3.6 (-4.3- -3.0) | 11,821 | -6.0 (-6.5- -5.5) | 60,516 | -4.5 (-4.7- -4.3) | 36,997 | -2.8 (-3.1- -2.6) | 73,134 | -4.2 (-4.4- -4.0) |
|  | **November** | 207,432 | -3.0 (-3.1- -2.9) | 28 | 46.9 (29.8- 66.4) | 1,409 | 28.2 (26.1- 30.4) | 8,602 | -2.2 (-2.8- -1.6) | 13,037 | -5.7 (-6.1- -5.2) | 66,604 | -3.5 (-3.7- -3.2) | 40,257 | -1.8 (-2.1- -1.5) | 77,495 | -3.1 (-3.3- -2.9) |
|  | **December** | 217,098 | -2.8 (-2.9- -2.6) | 29 | 15.4 (2.6- 29.7) | 1,617 | 34.5 (32.4- 36.6) | 9,253 | -1.5 (-2.0- -0.9) | 13,734 | -5.1 (-5.5- -4.6) | 70,219 | -3.2 (-3.4- -3.0) | 42,162 | -1.6 (-1.9- -1.4) | 80,084 | -3.1 (-3.3- -2.9) |
| **2022** | **January** | 213,372 | -1.7 (-1.8- -1.6) | 27 | 17.0 (2.5- 33.5) | 1,543 | 39.3 (36.8- 41.9) | 9,150 | -0.2 (-0.9- 0.4) | 13,376 | -3.9 (-4.4- -3.4) | 68,978 | -1.0 (-1.2- -0.7) | 41,251 | 0.1 (-0.2- 0.4) | 79,047 | -3.2 (-3.4- -3.0) |
|  | **February** | 198,512 | -0.4 (-0.5- -0.2) | 19 | -9.2 (-22.2- 6.0) | 1,384 | 39.3 (36.6- 42.1) | 8,536 | 2.0 (1.3- 2.7) | 12,023 | -3.6 (-4.1- -3.0) | 63,965 | 0.2 (-0.0- 0.5) | 37,708 | 0.7 (0.4- 1.0) | 74,877 | -1.4 (-1.6- -1.1) |
|  | **March** | 185,566 | 0.1 (0.0- 0.3) | 17 | 25.1 (8.1- 44.9) | 1,242 | 45.8 (42.9- 48.8) | 7,973 | 5.8 (5.0- 6.5) | 11,010 | -3.6 (-4.2- -3.1) | 59,099 | 0.4 (0.2- 0.7) | 34,799 | 1.4 (1.0- 1.7) | 71,426 | -0.9 (-1.2- -0.7) |
|  | **April** | 162,414 | -2.5 (-2.7- -2.4) | 23 | 14.7 (-1.0- 32.8) | 1,163 | 38.9 (36.0- 41.9) | 7,173 | 4.8 (4.0- 5.6) | 9,809 | -3.8 (-4.4- -3.2) | 51,681 | -1.7 (-1.9- -1.4) | 29,967 | -2.5 (-2.8- -2.1) | 62,598 | -4.3 (-4.5- -4.1) |
|  | **May** | 158,956 | -3.8 (-4.0- -3.7) | 22 | 15.3 (-0.7- 33.9) | 1,155 | 47.5 (44.4- 50.6) | 7,002 | 4.8 (4.0- 5.6) | 9,473 | -5.5 (-6.1- -4.9) | 49,917 | -3.8 (-4.1- -3.6) | 29,498 | -3.4 (-3.7- -3.0) | 61,889 | -5.2 (-5.4- -4.9) |
|  | **June** | 165,316 | -4.8 (-4.9- -4.7) | 23 | 38.9 (21.0- 59.3) | 1,200 | 47.4 (44.4- 50.5) | 7,274 | 4.0 (3.2- 4.7) | 9,683 | -7.2 (-7.7- -6.6) | 51,460 | -4.6 (-4.9- -4.4) | 30,899 | -4.7 (-5.0- -4.4) | 64,777 | -6.1 (-6.3- -5.8) |
|  | **July** | 171,640 | -5.4 (-5.5- -5.2) | 21 | -4.2 (-17.1- 10.6) | 1,161 | 41.4 (38.6- 44.3) | 7,463 | 2.3 (1.6- 3.0) | 9,998 | -7.1 (-7.7- -6.6) | 53,624 | -5.1 (-5.4- -4.9) | 32,352 | -4.7 (-5.0- -4.4) | 67,021 | -6.9 (-7.1- -6.7) |
|  | **August** | 174,242 | -4.7 (-4.9- -4.6) | 18 | -17.1 (-28.8- -3.4) | 1,148 | 38.2 (35.4- 41.0) | 7,636 | 2.9 (2.1- 3.6) | 10,067 | -7.9 (-8.4- -7.4) | 54,354 | -5.2 (-5.4- -5.0) | 32,943 | -3.4 (-3.7- -3.1) | 68,076 | -5.7 (-5.9- -5.5) |
|  | **September** | 180,926 | -4.5 (-4.6- -4.3) | 32 | 55.0 (35.6- 77.1) | 1,254 | 41.1 (38.3- 44.0) | 7,991 | 2.6 (1.9- 3.3) | 10,373 | -8.1 (-8.6- -7.6) | 56,908 | -4.4 (-4.6- -4.1) | 34,320 | -3.1 (-3.4- -2.8) | 70,048 | -5.9 (-6.1- -5.7) |
|  | **October** | 194,965 | -3.7 (-3.9- -3.6) | 30 | 36.9 (19.7- 56.5) | 1,475 | 43.3 (40.6- 46.1) | 8,824 | 5.0 (4.3- 5.8) | 11,345 | -8.2 (-8.6- -7.7) | 61,745 | -3.3 (-3.5- -3.1) | 37,300 | -2.3 (-2.6- -2.0) | 74,246 | -5.5 (-5.7- -5.3) |
|  | **November** | 212,466 | -2.0 (-2.1- -1.8) | 33 | 95.6 (72.0- 122.4) | 1,727 | 48.3 (45.6- 51.0) | 9,904 | 9.4 (8.7- 10.2) | 12,749 | -6.1 (-6.6- -5.6) | 68,272 | -1.9 (-2.1- -1.6) | 40,762 | -0.5 (-0.8- -0.2) | 79,019 | -3.9 (-4.2- -3.7) |
|  | **December** | 222,457 | -1.7 (-1.8- -1.5) | 35 | 74.2 (54.6- 96.4) | 1,928 | 48.2 (45.7- 50.9) | 10,458 | 8.7 (8.0- 9.4) | 13,492 | -4.5 (-5.0- -4.1) | 71,963 | -1.6 (-1.8- -1.4) | 42,854 | -0.4 (-0.7- -0.1) | 81,727 | -3.6 (-3.8- -3.4) |

**Supplementary Table 2:** Monthly number of AD consumers in the observation period (2020-2022) for each subpopulation, and variations from the 2017-2019 trend expressed as a percentage.

|  |  | **Female** | | **Male** | | **Foreign nationality** | | **Rural** | | **Small town** | | **City** | |
| --- | --- | --- | --- | --- | --- | --- | --- | --- | --- | --- | --- | --- | --- |
| **Year** | **Month** | **N** | **(IRR-1)% (CI 95%)** | **N** | **(IRR-1)% (CI 95%)** | **N** | **(IRR-1)% (CI 95%)** | **N** | **(IRR-1)% (CI 95%)** | **N** | **(IRR-1)% (CI 95%)** | **N** | **(IRR-1)% (CI 95%)** |
| **2020** | **January** | 150,435 | -0.9 (-1.0- -0.7) | 65,242 | -0.8 (-1.0- -0.6) | 6,481 | -2.7 (-3.4- -2.1) | 66,664 | -0.8 (-1.0- -0.6) | 70,709 | -0.6 (-0.8- -0.4) | 77,353 | -1.0 (-1.2- -0.8) |
|  | **February** | 137,260 | -2.0 (-2.1- -1.8) | 59,265 | -1.9 (-2.2- -1.7) | 5,963 | -5.2 (-5.9- -4.5) | 60,678 | -2.1 (-2.3- -1.9) | 64,572 | -1.5 (-1.7- -1.3) | 70,434 | -2.3 (-2.5- -2.1) |
|  | **March** | 125,741 | -0.9 (-1.1- -0.8) | 54,371 | -1.3 (-1.5- -1.1) | 5,430 | -6.5 (-7.2- -5.8) | 55,391 | -1.9 (-2.1- -1.7) | 59,307 | -0.4 (-0.6- -0.2) | 64,662 | -0.8 (-1.0- -0.6) |
|  | **April** | 109,418 | -2.2 (-2.4- -2.1) | 47,417 | -1.9 (-2.2- -1.7) | 4,612 | -14.0 (-14.7- -13.4) | 48,280 | -2.6 (-2.9- -2.4) | 51,568 | -1.1 (-1.4- -0.9) | 56,330 | -2.6 (-2.8- -2.4) |
|  | **May** | 106,499 | -2.9 (-3.1- -2.8) | 46,118 | -3.2 (-3.4- -2.9) | 4,554 | -14.7 (-15.4- -14.0) | 47,280 | -3.2 (-3.4- -2.9) | 50,253 | -2.2 (-2.4- -1.9) | 54,417 | -3.5 (-3.8- -3.3) |
|  | **June** | 112,799 | -3.3 (-3.5- -3.2) | 48,669 | -2.9 (-3.1- -2.6) | 4,878 | -12.2 (-12.9- -11.5) | 49,556 | -3.5 (-3.7- -3.3) | 53,172 | -2.2 (-2.4- -2.0) | 58,044 | -3.8 (-4.0- -3.6) |
|  | **July** | 121,353 | -2.3 (-2.4- -2.2) | 52,303 | -2.3 (-2.5- -2.1) | 5,323 | -9.2 (-9.8- -8.5) | 53,161 | -2.8 (-3.0- -2.6) | 57,031 | -1.5 (-1.8- -1.3) | 62,730 | -2.6 (-2.8- -2.4) |
|  | **August** | 120,449 | -2.2 (-2.3- -2.1) | 51,843 | -2.0 (-2.2- -1.8) | 5,234 | -8.0 (-8.6- -7.3) | 53,302 | -2.5 (-2.7- -2.3) | 56,684 | -1.3 (-1.5- -1.1) | 61,578 | -2.6 (-2.8- -2.4) |
|  | **September** | 125,590 | -2.3 (-2.4- -2.1) | 54,139 | -2.1 (-2.3- -1.9) | 5,474 | -6.1 (-6.8- -5.4) | 55,828 | -2.5 (-2.7- -2.3) | 59,284 | -1.7 (-1.9- -1.5) | 63,822 | -2.5 (-2.7- -2.3) |
|  | **October** | 136,920 | -1.3 (-1.5- -1.2) | 58,882 | -1.1 (-1.3- -0.9) | 5,954 | -8.0 (-8.7- -7.4) | 60,569 | -1.3 (-1.5- -1.1) | 64,604 | -0.4 (-0.6- -0.2) | 69,773 | -1.9 (-2.1- -1.8) |
|  | **November** | 146,598 | 0.9 (0.7- 1.0) | 62,864 | 0.7 (0.5- 0.9) | 6,385 | -6.9 (-7.5- -6.2) | 64,467 | 0.4 (0.2- 0.6) | 69,120 | 1.5 (1.3- 1.7) | 74,971 | 0.5 (0.3- 0.7) |
|  | **December** | 148,336 | -3.1 (-3.2- -3.0) | 64,159 | -3.5 (-3.6- -3.3) | 6,483 | -11.0 (-11.5- -10.4) | 64,535 | -5.0 (-5.1- -4.8) | 70,128 | -2.4 (-2.6- -2.2) | 76,995 | -2.2 (-2.4- -2.1) |
| **2021** | **January** | 144,457 | -5.1 (-5.3- -5.0) | 62,751 | -5.2 (-5.4- -5.0) | 6,323 | -14.6 (-15.2- -14.0) | 62,610 | -8.0 (-8.2- -7.8) | 68,504 | -4.1 (-4.3- -3.9) | 75,330 | -3.4 (-3.6- -3.2) |
|  | **February** | 136,178 | -0.8 (-1.0- -0.7) | 58,791 | -1.5 (-1.7- -1.3) | 6,080 | -11.6 (-12.2- -10.9) | 59,254 | -2.9 (-3.1- -2.7) | 64,496 | 0.2 (-0.0- 0.4) | 70,476 | -0.4 (-0.6- -0.2) |
|  | **March** | 129,866 | 1.9 (1.7- 2.0) | 55,812 | 0.8 (0.6- 1.1) | 6,001 | -12.0 (-12.7- -11.4) | 56,624 | 0.4 (0.1- 0.6) | 61,256 | 2.3 (2.1- 2.6) | 67,060 | 1.8 (1.6- 2.1) |
|  | **April** | 114,386 | -0.6 (-0.8- -0.5) | 49,271 | -0.7 (-0.9- -0.4) | 5,515 | -12.8 (-13.5- -12.1) | 49,796 | -2.4 (-2.7- -2.2) | 54,092 | 1.1 (0.9- 1.4) | 59,072 | -0.8 (-1.0- -0.5) |
|  | **May** | 109,853 | -2.1 (-2.3- -2.0) | 47,730 | -2.2 (-2.4- -1.9) | 5,290 | -12.7 (-13.4- -12.0) | 47,991 | -4.1 (-4.3- -3.9) | 52,261 | -0.3 (-0.6- -0.1) | 56,677 | -2.2 (-2.4- -2.0) |
|  | **June** | 114,444 | -4.4 (-4.5- -4.2) | 48,976 | -4.8 (-5.0- -4.6) | 5,351 | -16.5 (-17.2- -15.8) | 49,478 | -6.0 (-6.3- -5.8) | 54,070 | -2.8 (-3.0- -2.5) | 59,174 | -4.8 (-5.0- -4.6) |
|  | **July** | 120,471 | -4.1 (-4.3- -4.0) | 51,278 | -5.1 (-5.4- -4.9) | 5,527 | -16.1 (-16.8- -15.5) | 52,065 | -5.8 (-6.0- -5.6) | 56,978 | -2.8 (-3.0- -2.6) | 61,992 | -4.8 (-5.0- -4.6) |
|  | **August** | 120,437 | -3.5 (-3.7- -3.4) | 51,250 | -4.9 (-5.1- -4.7) | 5,404 | -17.1 (-17.7- -16.4) | 52,518 | -5.3 (-5.5- -5.0) | 56,985 | -2.2 (-2.5- -2.0) | 61,475 | -4.3 (-4.5- -4.1) |
|  | **September** | 124,685 | -4.1 (-4.2- -3.9) | 52,954 | -5.5 (-5.7- -5.3) | 5,563 | -16.4 (-17.0- -15.7) | 54,240 | -6.2 (-6.4- -6.0) | 58,740 | -3.6 (-3.9- -3.4) | 63,915 | -3.8 (-4.0- -3.6) |
|  | **October** | 134,271 | -3.7 (-3.8- -3.5) | 57,077 | -5.0 (-5.2- -4.7) | 5,968 | -18.0 (-18.6- -17.4) | 58,327 | -5.9 (-6.1- -5.7) | 63,313 | -2.9 (-3.2- -2.7) | 68,915 | -3.5 (-3.7- -3.3) |
|  | **November** | 145,345 | -2.6 (-2.8- -2.5) | 62,087 | -3.8 (-4.0- -3.6) | 6,497 | -18.0 (-18.6- -17.5) | 63,206 | -4.4 (-4.6- -4.2) | 68,895 | -1.7 (-1.9- -1.5) | 74,482 | -2.9 (-3.1- -2.7) |
|  | **December** | 152,030 | -2.2 (-2.3- -2.0) | 65,068 | -4.0 (-4.2- -3.8) | 6,763 | -18.8 (-19.4- -18.3) | 65,924 | -4.5 (-4.7- -4.3) | 72,203 | -1.1 (-1.3- -0.9) | 78,051 | -2.8 (-3.0- -2.6) |
| **2022** | **January** | 149,481 | -1.0 (-1.2- -0.8) | 63,891 | -3.1 (-3.4- -2.9) | 6,675 | -19.0 (-19.7- -18.4) | 64,953 | -3.2 (-3.5- -3.0) | 70,863 | 0.3 (0.0- 0.5) | 76,639 | -1.9 (-2.2- -1.7) |
|  | **February** | 139,139 | 0.3 (0.1- 0.4) | 59,373 | -1.8 (-2.0- -1.5) | 6,430 | -18.9 (-19.5- -18.2) | 60,245 | -2.0 (-2.2- -1.7) | 65,855 | 1.2 (0.9- 1.4) | 71,532 | -0.3 (-0.6- -0.1) |
|  | **March** | 130,006 | 0.8 (0.6- 0.9) | 55,560 | -1.2 (-1.4- -0.9) | 6,242 | -17.6 (-18.3- -16.9) | 56,285 | -1.8 (-2.0- -1.5) | 61,373 | 1.8 (1.6- 2.1) | 67,073 | 0.4 (0.2- 0.6) |
|  | **April** | 113,483 | -2.4 (-2.6- -2.2) | 48,931 | -2.8 (-3.0- -2.5) | 5,602 | -22.3 (-23.0- -21.6) | 49,243 | -4.8 (-5.0- -4.5) | 53,610 | -0.8 (-1.0- -0.5) | 58,823 | -2.2 (-2.4- -1.9) |
|  | **May** | 110,996 | -3.6 (-3.8- -3.4) | 47,960 | -4.2 (-4.5- -4.0) | 5,461 | -23.7 (-24.4- -23.1) | 48,159 | -6.0 (-6.3- -5.7) | 52,464 | -2.5 (-2.7- -2.2) | 57,600 | -3.2 (-3.4- -2.9) |
|  | **June** | 115,626 | -4.6 (-4.7- -4.4) | 49,690 | -5.2 (-5.4- -4.9) | 5,661 | -22.9 (-23.6- -22.2) | 50,076 | -6.5 (-6.8- -6.3) | 54,526 | -2.9 (-3.2- -2.7) | 59,953 | -5.0 (-5.2- -4.8) |
|  | **July** | 120,141 | -4.9 (-5.1- -4.8) | 51,499 | -6.2 (-6.5- -6.0) | 5,817 | -22.5 (-23.2- -21.9) | 51,958 | -7.0 (-7.2- -6.7) | 56,750 | -3.6 (-3.8- -3.3) | 62,118 | -5.7 (-5.9- -5.4) |
|  | **August** | 122,091 | -4.3 (-4.4- -4.1) | 52,151 | -5.7 (-5.9- -5.4) | 5,768 | -23.0 (-23.6- -22.4) | 53,030 | -6.8 (-7.0- -6.5) | 57,925 | -2.5 (-2.7- -2.2) | 62,474 | -5.0 (-5.2- -4.8) |
|  | **September** | 126,615 | -4.2 (-4.4- -4.1) | 54,311 | -4.9 (-5.2- -4.7) | 6,020 | -21.1 (-21.8- -20.5) | 54,894 | -7.2 (-7.4- -7.0) | 60,063 | -2.6 (-2.9- -2.4) | 65,148 | -3.7 (-3.9- -3.5) |
|  | **October** | 136,513 | -3.5 (-3.6- -3.3) | 58,452 | -4.2 (-4.4- -4.0) | 6,499 | -22.4 (-23.0- -21.8) | 59,026 | -6.0 (-6.3- -5.8) | 64,786 | -1.7 (-2.0- -1.5) | 70,281 | -3.5 (-3.7- -3.3) |
|  | **November** | 148,761 | -1.4 (-1.6- -1.3) | 63,705 | -3.1 (-3.3- -2.8) | 7,102 | -21.3 (-21.9- -20.7) | 64,304 | -4.4 (-4.6- -4.1) | 70,563 | 0.3 (0.1- 0.6) | 76,655 | -2.0 (-2.2- -1.7) |
|  | **December** | 155,473 | -1.3 (-1.4- -1.1) | 66,984 | -2.4 (-2.6- -2.2) | 7,399 | -21.0 (-21.6- -20.4) | 67,212 | -4.1 (-4.3- -3.8) | 73,871 | 0.4 (0.2- 0.6) | 80,365 | -1.4 (-1.6- -1.2) |

**Supplementary Table 3:** Monthly incidence of new AD consumers and DDDs/1000 inhabitants and variation from the 2017-2019 trend expressed in (IRR-1)% among age groups 12-19 and 20-34, from 2020 to 2022.

|  |  | **12-19** | | | | **20-34** | | | |
| --- | --- | --- | --- | --- | --- | --- | --- | --- | --- |
| **Years** | **Month** | **New AD consumers** | **(IRR-1)% (CI 95%)** | **DDDx 1000** | **(IRR-1)% (CI 95%)** | **New AD consumers** | **(IRR-1)% (CI 95%)** | **DDDx 1000** | **(IRR-1)% (CI 95%)** |
| **2020** | **January** | 100 | -10.41 (-30.68- 15.79) | 95 | 6.12 (-23.1- 46.43) | 739 | 1.17 (-11.90- 16.18) | 458 | 1.88 (-25.2- 38.84) |
|  | **February** | 108 | -7.39 (-27.94- 19.03) | 96 | 6.99 (-22.6- 47.86) | 676 | -9.40 (-21.48- 4.53) | 413 | -0.36 (-27.7- 37.35) |
|  | **March** | 70 | -47.60 (-60.50- -30.48) | 100 | 1.96 (-25.5- 39.59) | 569 | -31.47 (-40.63- -20.90) | 469 | 2.66 (-24.7- 39.94) |
|  | **April** | 57 | -53.99 (-66.12- -37.50) | 97 | -0.79 (-27.9- 36.44) | 516 | -30.90 (-40.38- -19.90) | 407 | -3.34 (-29.5- 32.49) |
|  | **May** | 49 | -55.61 (-67.90- -38.62) | 90 | -20.7 (-42.3- 8.91) | 542 | -27.41 (-37.24- -16.06) | 402 | -16.5 (-38.9- 13.93) |
|  | **June** | 80 | -11.94 (-33.53- 16.66) | 98 | -12.5 (-36.2- 19.89) | 619 | -17.55 (-28.54- -4.85) | 441 | -0.62 (-27.4- 36.08) |
|  | **July** | 78 | -12.17 (-33.90- 16.72) | 104 | -6.74 (-31.4- 26.78) | 700 | -14.39 (-25.47- -1.67) | 484 | -2.76 (-28.5- 32.31) |
|  | **August** | 66 | -8.29 (-32.30- 24.21) | 84 | -9.66 (-34.9- 25.34) | 515 | -17.91 (-29.22- -4.79) | 397 | -8.97 (-33.4- 24.37) |
|  | **September** | 108 | 1.92 (-20.93- 31.38) | 101 | -10.4 (-34.4- 22.28) | 668 | -10.12 (-22.04- 3.62) | 451 | 0.15 (-26.8- 37.07) |
|  | **October** | 157 | 6.23 (-14.72- 32.34) | 114 | -1.84 (-27.4- 32.69) | 770 | -12.39 (-23.53- 0.37) | 471 | -2.45 (-28.4- 32.85) |
|  | **November** | 168 | 3.79 (-16.20- 28.55) | 114 | -1.64 (-27.4- 33.24) | 794 | -6.12 (-18.19- 7.74) | 444 | -5.29 (-30.8- 29.63) |
|  | **December** | 182 | 0.48 (-18.43- 23.78) | 122 | -6.44 (-30.5- 25.89) | 768 | -12.72 (-23.88- 0.08) | 445 | -4.88 (-30.2- 29.65) |
| **2021** | **January** | 122 | 5.07 (-19.06- 36.40) | 98 | -10.1 (-35.9- 26.04) | 698 | -6.23 (-19.34- 9.00) | 444 | -6.55 (-32.9- 30.13) |
|  | **February** | 143 | 12.35 (-12.67- 44.53) | 101 | -1.40 (-29.9- 38.61) | 759 | -6.28 (-19.45- 9.05) | 410 | -2.95 (-31.2- 36.99) |
|  | **March** | 141 | -8.19 (-28.32- 17.59) | 129 | 7.95 (-21.5- 48.53) | 913 | -4.74 (-17.48- 9.97) | 510 | 5.69 (-24.0- 47.06) |
|  | **April** | 136 | -1.52 (-23.47- 26.72) | 126 | 13.25 (-18.2- 56.80) | 814 | -2.03 (-15.47- 13.55) | 481 | 8.15 (-22.6- 51.04) |
|  | **May** | 160 | 31.87 (3.30- 68.33) | 131 | 1.49 (-26.1- 39.41) | 721 | -12.86 (-24.90- 1.12) | 434 | -14.7 (-38.7- 18.84) |
|  | **June** | 120 | 18.28 (-9.59- 54.75) | 134 | 9.28 (-20.8- 50.72) | 709 | -14.92 (-26.78- -1.15) | 478 | 1.95 (-27.0- 42.42) |
|  | **July** | 121 | 25.77 (-3.89- 64.57) | 147 | 19.55 (-12.6- 63.43) | 719 | -18.73 (-29.91- -5.76) | 543 | 3.51 (-25.4- 43.66) |
|  | **August** | 120 | 46.78 (11.65- 92.98) | 118 | 14.79 (-17.4- 59.59) | 628 | -11.56 (-24.11- 3.07) | 474 | 3.06 (-26.0- 43.47) |
|  | **September** | 140 | 18.36 (-8.35- 52.85) | 134 | 8.44 (-21.2- 49.21) | 696 | -17.66 (-29.19- -4.27) | 548 | 15.23 (-17.4- 60.72) |
|  | **October** | 168 | 7.22 (-15.34- 35.79) | 152 | 16.20 (-14.7- 58.28) | 803 | -14.77 (-26.34- -1.39) | 538 | 5.66 (-23.9- 46.75) |
|  | **November** | 241 | 29.81 (4.33- 61.51) | 158 | 20.13 (-11.9- 63.79) | 965 | -3.22 (-16.15- 11.69) | 493 | -0.34 (-28.6- 39.12) |
|  | **December** | 315 | 55.44 (26.15- 91.54) | 194 | 40.52 (3.88- 90.08) | 885 | -10.00 (-22.10- 3.99) | 524 | 6.09 (-23.6- 47.40) |
| **2022** | **January** | 166 | 30.20 (-0.53- 70.42) | 131 | 13.76 (-20.1- 61.98) | 791 | -6.75 (-20.60- 9.51) | 816 | 62.88 (14.41- 131.9) |
|  | **February** | 210 | 48.88 (14.79- 93.09) | 175 | 55.92 (10.29- 120.4) | 859 | -4.02 (-18.40- 12.88) | 770 | 72.87 (19.95- 149.1) |
|  | **March** | 204 | 19.00 (-7.90- 53.77) | 257 | 96.37 (41.56- 172.4) | 960 | -10.15 (-23.13- 5.02) | 987 | 93.92 (36.26- 176.0) |
|  | **April** | 203 | 36.79 (5.60- 77.20) | 213 | 80.46 (28.73- 153.0) | 820 | -8.29 (-21.88- 7.65) | 818 | 74.51 (22.18- 149.3) |
|  | **May** | 179 | 30.30 (0.06- 69.67) | 246 | 68.60 (21.75- 133.5) | 880 | -6.74 (-20.38- 9.24) | 975 | 81.80 (27.89- 158.5) |
|  | **June** | 154 | 36.28 (3.08- 80.18) | 235 | 68.67 (20.98- 135.2) | 790 | -14.52 (-27.23- 0.40) | 905 | 82.83 (28.01- 161.1) |
|  | **July** | 133 | 29.95 (-2.45- 73.11) | 279 | 114.9 (55.14- 197.6) | 746 | -20.39 (-32.21- -6.50) | 844 | 52.40 (7.20- 116.7) |
|  | **August** | 118 | 26.71 (-5.82- 70.46) | 213 | 90.04 (35.63- 166.3) | 713 | -12.76 (-25.85- 2.64) | 713 | 46.92 (3.04- 109.5) |
|  | **September** | 154 | 16.68 (-11.38- 53.64) | 308 | 126.5 (63.64- 213.5) | 784 | -16.17 (-28.67- -1.48) | 735 | 46.53 (2.47- 109.5) |
|  | **October** | 219 | 26.81 (-1.37- 63.04) | 268 | 85.65 (34.35- 156.5) | 902 | -13.01 (-25.65- 1.78) | 861 | 60.10 (12.56- 127.7) |
|  | **November** | 260 | 25.60 (-1.55- 60.24) | 320 | 122.7 (61.40- 207.3) | 1,027 | -6.33 (-19.85- 9.46) | 962 | 84.31 (29.12- 163.1) |
|  | **December** | 272 | 23.97 (-2.63- 57.84) | 440 | 191.2 (112.2- 299.7) | 957 | -11.00 (-23.88- 4.06) | 846 | 62.31 (14.07- 130.9) |
